# Mr. Smee on the Potato Plant

**Published:** 1847-04

**Authors:** 


Art. III.—*The Potato Plant, its Uses and Properties : together with the Cause of the Present Malady. The Extension of that Disease to other Plants, the Question of Famine arising therefrom, and the best Means of averting that Calamity.* By Alfred Smee, f.r.s., Surgeon to the Bank of England, &c.—*London,* 1846. 8vo, pp. 174. With 10 Plates.

Our author's promises as set forth in this title-page are large enough, and form a striking contrast to his amount of performance. Everybody knows that of the thousand-and-one hypotheses which have been proposed to account for the fearful infliction that has fallen upon the cultivators of the potato, each singly, however specious it appeared at first sight, has been proved to be deficient in that universality of application, which could alone warrant its reception as the *vera causa.* Nothing daunted, however, by the failure of his predecessors, Mr. Smee plunges boldly into the investigation; convinced that a medical man is the one to make the discovery for which vegetable physiologists and agriculturists have vainly sought, and that his own qualifications for the search peculiarly marked him out as the fortunate benefactor of mankind, whose name should be handed down to succeeding generations as long as the potato shall be cultivated for the food of man or beast. "The business of a surgeon," he says, "is essentially locomotive, and his duties are practised over an extensive space. It frequently happens that I have had to traverse London in two or even more directions in a single day, which circumstance has given me abundant opportunities of making my observations in different localities." We should like to see these vast potato-fields which Mr. Smee is so clever in perambulating between the extremities of our smoky metropolis, where we thought that nothing grew but houses and "humans." But his researches have not been confined to London alone. They have extended even as far as Clapton, which (we may inform our country readers) is no less than four miles from the Bank. "During the summer months," he informs us, "I was living at Springfield, Upper Clapton, where I had the advantage of a large garden, wherein were several plots of potatoes, which I was in the habit of observing the first thing in the morning, again on my return from London, and frequently the last thing at night. In the neighbourhood, moreover, were larger potato grounds, where I used to enjoy the air and study the disease in the evening; and it has curiously happened, that I have made my observations on the potato-plant in the same garden in which I considered the experiments for my former work on Electro-Metallurgy;" a coincidence which will doubtless impress the public with additional confidence in the author's results.

Finding a few diseased potato-plants at Clapton, covered with aphides,

Mr. Smee seems to have at once concluded that he had found out the true secret of the disease; which is just as if he had argued from the presence of lice upon the persons of a dozen or two of hospital-patients affected with pneumonia or rheumatism, that they were the cause of the malady. We have carefully searched his treatise for that collection of evidence on the subject which is necessary to establish anything like a satisfactory relation of cause and effect; but we have been utterly disappointed. No locality is referred to as furnishing the aphides, save the potato-grounds at Clapton; no results are adduced of inquiries made in distant parts of the country; no proof is given that aphides placed upon healthy potato-plants, and allowed to propagate there, will produce *the* potato-disease in question. We cannot too strongly express our surprise, that a gentleman of Mr. Smee's scientific attainments should have ventured, after all the experience of former failures in accounting satisfactorily for the disease, to make such confident assertions regarding its cause upon so meagre an amount of evidence; when a very short delay would have been sufficient to enable him to procure such confirmatory testimony as the nature of the case admitted from every quarter of the kingdom, the attention of almost everybody being alive to the subject, and any new theory being caught at with avidity. We are not passing judgment upon the merits of the doctrine itself, for we do not consider ourselves competent to do so. The question is entirely one of evidence; since the *à priori* probability seems to us rather in Mr. Smee's favour than against him. And we understand that *since* the publication of his book, he has collected a large amount of evidence which he ought to have got together previously. But our sole concern is at present with the Treatise itself; and whilst we admit that it contains much valuable and interesting matter bearing upon the question at issue, we must repeat that it has not the slightest claim to be regarded as a justification of its author's assumptions.

